# Analysis of influencing factors and urban–rural difference decomposition of mental health literacy among residents in Chongqing, Southwest China: a large-scale multistage random sampling survey

**DOI:** 10.3389/fpubh.2026.1895974

**Published:** 2026-08-05

**Authors:** Deliang Kong, Jiyuan Zhong, Cuihong Li, Wenjun Wang, Hao Yang, Zixun Liu, Tianyi Yang, Xiaoping Cheng, Guoqing Jiang, Jianjun Luo

**Affiliations:** Chongqing Mental Health Center, Chongqing, China

**Keywords:** contribution decomposition, Fairlie decomposition analysis, mental health literacy, multistage random sampling, urban–rural differences

## Abstract

**Background:**

Mental health literacy (MHL), as a core issue in public health, is closely associated with population mental health status and the ability to cope with psychological problems. This study aimed to evaluate the level of MHL among residents in Chongqing, identify its influencing factors, and explore the sources of urban–rural differences in MHL.

**Methods:**

A stratified multistage random sampling method was used to conduct a questionnaire survey among 36,874 permanent residents in Chongqing in 2025. The National Mental Health Literacy Questionnaire (NMHLQ) was used to assess MHL attainment. *χ*^2^ tests, logistic regression, and urban–rural weighted logistic regression were used to identify the influencing factors of MHL among urban and rural residents. The generalized variance inflation factor (GVIF) was used to assess multicollinearity among variables. A weighted Fairlie nonlinear decomposition model was applied to quantify the contributions to urban–rural differences, and repeated Fairlie decomposition with adjusted sample coefficients was used as a sensitivity analysis. Stratified analyses were conducted to verify the robustness of the results. Statistical analyses were performed using SPSS 22.0, R 4.2.2, and Stata MP16.0.

**Results:**

The overall MHL attainment rate among residents was 32.21%, with urban residents showing a higher rate than rural residents (37.08% vs. 30.26%, *p* < 0.001). After weighting according to the urban–rural population structure, the overall attainment rate was 35.18%. Multivariable analysis showed that age, education level, income level, occupation, health behaviors, self-rated health (SRH), and chronic disease status were influencing factors of MHL among urban and rural residents. Urban–rural weighted logistic regression showed that urban residents had higher odds of MHL attainment than rural residents (OR = 1.36, 95%CI: 1.29–1.43, *p* < 0.001). After adjustment for covariates in the stratified analyses, urban residents still had higher odds of MHL attainment. Weighted Fairlie decomposition showed that 0.0442 (64.83%) of the difference could not be explained by the observed covariates, which may reflect unmeasured structural urban–rural differences or other unobserved factors. A total of 0.0240 (35.17%) of the difference was attributable to observed factors, and the contribution rates of occupation, education level, monthly household income, and regional distribution were 27.01, 26.55, 12.90, and −35.13%, respectively. Repeated Fairlie decomposition with adjusted sample coefficients showed relatively stable results.

**Conclusion:**

The MHL attainment rate among residents in Chongqing was at a moderately high level; however, significant urban–rural disparities remained. Occupation, education level, and household economic conditions were the main sources of these differences. Greater attention should be paid to rural residents and populations with low education levels, low income, and poor health status. It is recommended to integrate primary healthcare, community education, and digital communication resources to promote equitable improvement in MHL.

## Graphical abstract

**Figure:**
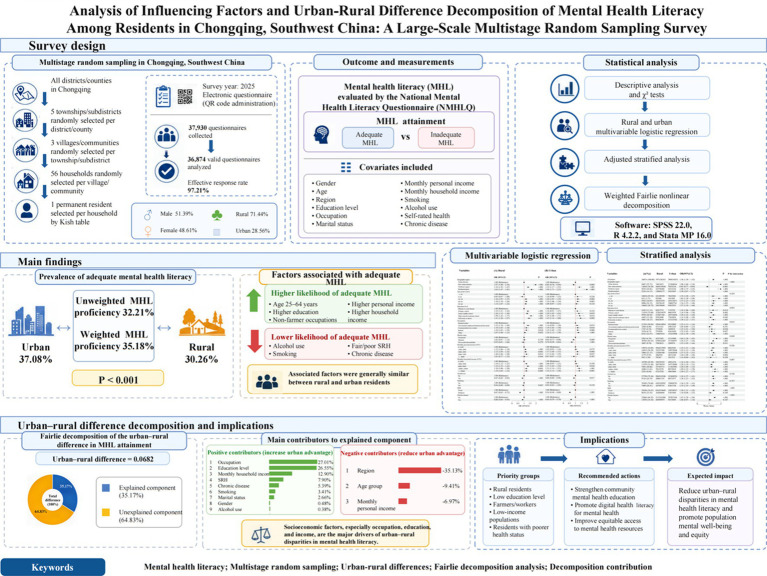
SRH, self-rated health; MHL, mental health literacy; NMHLQ, National Mental Health Literacy Questionnaire.

## Introduction

Mental health problems have become an important issue in the global burden of disease and public health governance. The World Health Organization has pointed out that mental disorders are widespread worldwide, and a substantial gap still exists between the demand for and supply of mental health services (1). The Global Burden of Disease Study showed that approximately 970 million people worldwide were living with mental disorders in 2019, with anxiety disorders and depressive disorders being the most common types (2, 3). A national epidemiological survey of mental disorders in China showed that the lifetime prevalence of any mental disorder among adults was 16.6%, and the 12-month prevalence was 9.3%, with anxiety disorders and mood disorders being the major disease types (4). Mental health literacy (MHL), as an important capability for the public to recognize, understand, prevent, and cope with mental health problems, is regarded as an important foundation for promoting early identification, reducing stigma, improving help-seeking behaviors, and enhancing service utilization (5–8). Previous studies have shown that MHL is closely associated with professional help-seeking attitudes, self-stigma, intention to use mental health services, and outcomes such as depression, anxiety, and sleep quality (6–10). Therefore, accurately evaluating the level of MHL among residents at the population level and identifying its influencing factors are of great significance for promoting mental health promotion, optimizing mental health education strategies, and improving the accessibility of primary mental health services.

There are still differences between urban and rural residents in China in terms of socioeconomic development, occupational structure, healthcare service provision, and access to health information, which may affect the level of MHL and service utilization. Previous studies have shown that inequities exist in healthcare service utilization between urban and rural residents in China, and that factors such as income, age, chronic disease status, and medical insurance are closely associated with differences in health service utilization (11). Rural older adults still face limitations in service accessibility, medical expenditure burden, and healthcare service experience (12). Studies among older Chinese populations have found that the prevalence level and changing trends of depressive symptoms differ between rural and urban older adults, and that factors such as education level, self-rated health (SRH), sleep quality, and life satisfaction are associated with urban–rural differences (13). Studies on urban–rural differences in anxiety symptoms among Chinese adults have also shown that anxiety levels are higher among rural adults than among urban adults, and that a substantial proportion of the differences can be explained by sociodemographic and social support factors (14). A survey of urban and rural residents in Guangdong Province showed that the MHL attainment rate was 13.6% among urban residents and 8.6% among rural residents (15), suggesting that disparities exist between urban and rural populations in terms of mental health knowledge and literacy. Therefore, examining differences in MHL and their sources from the perspective of urban–rural equity may help identify priorities for mental health promotion from an equity perspective.

In recent years, the Chinese government has continuously included MHL as an important monitoring indicator for mental health promotion actions. The Healthy China Action (2019–2030) clearly proposed targets for improving the level of MHL among residents, stating that the level of MHL among residents should increase to 20% by 2022 and 30% by 2030, and that a unified survey instrument and monitoring system should be used to promote the understanding of population MHL status across regions (16, 17). Chongqing is located in Southwest China and is a municipality directly under the central government in western China. Its jurisdiction includes urban districts, new urban districts, the northeast region, and the southeast region, and it is characterized by the coexistence of large cities, large rural areas, mountainous areas, and reservoir areas. According to the 2024 Chongqing Statistical Bulletin, the permanent resident population at the end of the year was 31.9047 million, including 23.0149 million urban residents and 8.8898 million rural residents, with an urbanization rate of 72.14%; the population aged 60 years and above was 8.0113 million, accounting for 25.11% of the total population (18). The large permanent resident population, ongoing urbanization process, and aging population may influence the MHL of Chongqing residents through factors such as the urban–rural spatial structure, population age composition, and primary healthcare service conditions. Previous studies have shown that self-rated health (SRH) is closely associated with mortality risk, chronic disease burden, and psychosocial factors (19, 20). Longitudinal studies based on middle-aged and older Chinese populations have also shown that depressive symptoms and chronic disease burden share similar changing trends, and that poorer SRH, insufficient sleep, and functional limitations are associated with higher levels of depressive symptoms and chronic disease burden (21). However, existing studies on MHL among residents in Southwest China remain relatively limited, particularly studies based on large-scale citywide community surveys that comprehensively incorporate demographic characteristics, socioeconomic factors, health behaviors, and health status factors while explaining the sources of urban–rural differences. In recent years, the Fairlie decomposition model has been widely used in research fields such as health inequality, mental health, and eHealth literacy, providing an effective method for exploring the causes of health-related inequalities between different populations and related variables (22–24). Based on this, relying on the national resident MHL survey task, this study conducted a multistage random sampling survey across the whole municipality of Chongqing to analyze the level of MHL attainment and its influencing factors among urban and rural residents, and applied a weighted Fairlie nonlinear decomposition model to evaluate the contributions to urban–rural differences in MHL attainment, aiming to provide evidence for identifying key intervention populations, optimizing stratified health promotion strategies, and promoting equity in mental health services.

## Methods

### Study design and data collection

The data for this study were derived from the 2025 Chongqing Residents’ Mental Health Literacy Survey conducted in Chongqing, Southwest China. This survey was implemented simultaneously across all districts and counties of Chongqing as part of work related to residents’ mental health promotion and MHL monitoring. To ensure that the survey participants met the study objectives and to improve data quality, this study further established inclusion and exclusion criteria during sampling and formal investigation. The inclusion criteria were permanent residents who lived in Chongqing during the survey period, were able to understand the questionnaire content, had the ability to complete the electronic questionnaire independently or under the guidance of investigators, and voluntarily participated in this study. The exclusion criteria included individuals with severe physical illness, those with cognitive impairment who were unable to complete the survey, and those who explicitly refused to participate.

The sample size estimation was based on the national and Chongqing target that the level of residents’ MHL would reach 30% by 2030 (17, 25). Considering that the attainment rate may differ across regions, urban and rural populations, and survey years, this study used *p* = 0.50 for calculation to obtain a relatively robust and conservative sample size. Under the conditions of *α* = 0.05 and an allowable error of *d* = 0.01, the initially estimated required sample size was approximately 9,604 participants. After correction for the multistage sampling design using a design effect of 2.0, the sample size was approximately 19,208 participants. Further considering invalid questionnaires, missing information, and other conditions, the minimum sample size was approximately 21,342 participants (26, 27). Meanwhile, according to the multistage sampling framework and the requirement for balanced sample coverage at each level, the planned survey sample size was 34,440 participants. A stratified multistage random sampling method was used to select participants step by step across all districts and counties of Chongqing. First, using the district- and county-level administrative units published in the Chongqing Statistical Yearbook as the sampling frame, townships or subdistricts within each district or county were numbered, and five townships or subdistricts were randomly selected. Second, three villages or communities were randomly selected from each selected township or subdistrict. Third, 56 households were randomly selected from each selected village or community. Finally, one permanent resident was randomly selected from each household using the Kish table method as the survey participant. This method has good randomness and operability in household surveys and helps reduce systematic bias in the participant selection process (28).

Considering that this study adopted a citywide multistage random sampling design, the survey focused on ensuring coverage of all districts and counties and primary-level survey units, as well as the standardized and effective implementation of the number of participants surveyed at each administrative level during the survey process. In the sampling frame, the number of townships and villages was greater than that of subdistricts and communities, and the surveyed sample included more rural residents than urban residents. Therefore, after obtaining the survey data, this study conducted weighted analysis based on the urban–rural population structure. The composition of the urban and rural permanent resident populations reported in the 2024 Statistical Communique of Chongqing on National Economic and Social Development was used as the population reference to calculate stratified weights for urban and rural residents (18). The questionnaire was administered electronically. Before the survey, Chongqing Mental Health Center provided standardized training for survey staff at all levels. During the formal survey, trained investigators guided respondents to complete the questionnaire by scanning a QR code with a mobile phone. A total of 37,930 questionnaires were collected. After excluding questionnaires with missing information or evidently low quality, 36,874 valid questionnaires were finally included, yielding an effective response rate of 97.21%. This study was reviewed and approved by the Ethics Committee of Chongqing Mental Health Center (Ethics Approval No. 2025–054), and all participants voluntarily participated after providing informed consent.

### Measurements and questionnaire

#### Demographic characteristics

This study collected information on participants’ demographic characteristics, socioeconomic status, health behaviors, and health-related information through a questionnaire. Demographic information mainly included gender, age, urban–rural residence, and regional distribution. Socioeconomic status mainly included education level, occupation, marital status, personal income, and household income. Health-related behaviors mainly included smoking and alcohol use. The above variables were organized according to classification methods commonly used in community resident health surveys and studies related to MHL. Urban–rural residence was determined using the questionnaire item, “Do you currently live in an urban or rural area?” Participants self-reported their status according to their actual place of residence, namely their current residence, rather than their household registration location. This approach is similar to the self-reported urban–rural classification adopted in studies by Nikoloski, Li, and others (29, 30). Because a large number of rural migrant workers may live in urban areas, and their household registration location may be inconsistent with their current place of residence, this study adopted self-reported current residence because it better reflects respondents’ current living environment, accessibility of primary healthcare services, and exposure to health information, while reducing misclassification caused by inconsistency between actual residence and household registration location. During survey implementation, uniformly trained investigators organized the survey in the selected villages or communities, and the survey address was required to be consistent with the respondent’s current main area of residence. For participants whose household registration location differed from their current residence, as well as for the floating population, urban–rural residence was recorded according to their actual place of residence at the time of the survey. Regional distribution was classified into urban districts, new urban districts, the northeast region, and the southeast region according to the administrative divisions and regional development characteristics of Chongqing (31). Smoking and alcohol use were determined according to the continuous behavioral habits reported by the respondents. Smoking was defined as smoking at least 1 cigarette per day for 6 months or longer, and alcohol use was defined as drinking alcohol at least once per week for 6 months or longer (32). Self-rated health (SRH) was assessed using the questionnaire item, “How do you think your health status has been over the past year?” and was categorized into “good” and “fair or poor” according to respondents’ answers (33). Chronic disease status was determined based on whether the respondent had ever been diagnosed by a physician with a relevant chronic disease. The questionnaire listed common types of chronic diseases and included an “other” option for respondents to provide additional information (34).

#### National Mental Health Literacy Questionnaire

This study used the National Mental Health Literacy Questionnaire (NMHLQ) to evaluate residents’ MHL. The questionnaire was developed by the Institute of Psychology, Chinese Academy of Sciences, based on the characteristics of mental health among Chinese residents and the needs of mental health promotion, and is an important survey instrument used in MHL monitoring among Chinese residents (35, 36). The NMHLQ consists of three parts: mental health knowledge judgment questions, self-assessment questions, and case analysis questions, with a total of 44 items. The mental health knowledge judgment section contains 20 items, each with three response options: “true,” “false,” and “do not know.” Correct answers are scored as 5 points, whereas incorrect answers or “do not know” responses are scored as 0 points, with a score range of 0–100. The self-assessment section contains 8 items, each scored from 1 to 4 points, with a total score range of 8–32. The case analysis section contains 16 items and mainly assesses residents’ ability to identify, cope with, and seek help for common mental illness scenarios, with a total score range of 0–40. According to the questionnaire scoring criteria, participants were classified as meeting the standard for MHL only if they simultaneously had a total score of ≥80 in the judgment section, ≥24 in the self-assessment section, and ≥28 in the case analysis section. Participants who failed to meet any one of the above criteria were classified as not meeting the standard for MHL. The NMHLQ has good psychometric performance. Previous studies have shown that the questionnaire has good internal consistency and structural validity, with a Cronbach’s coefficient of 0.870 and a Kaiser–Meyer–Olkin (KMO) coefficient of 0.891. It is also a nationally standardized and widely used questionnaire in surveys of MHL among Chinese residents (15, 35, 36).

### Statistical analysis

Data were organized, coded, transformed, and checked for logical consistency using Excel 2019. Univariate analysis and multivariable regression analysis were performed using SPSS 22.0 and R 4.2.2. Categorical variables were expressed as frequencies (*n*) and percentages (%). First, descriptive analyses were conducted to summarize the overall characteristics of the study participants, MHL attainment rate, and the weighted attainment rate, and to compare MHL attainment across different demographic, socioeconomic, and health-related variables. Second, *χ*^2^ tests were used to compare the distribution differences in MHL attainment between urban and rural residents across different variable strata. Furthermore, with MHL attainment as the dependent variable, logistic regression models were constructed separately in rural and urban samples. Variables that were statistically significant in the univariate analysis were included in the multivariable models to evaluate factors associated with MHL attainment among urban and rural residents, and the results were expressed as odds ratios (ORs) and 95% confidence intervals (95%CIs). To reduce the influence of the urban–rural composition difference between the sample and the overall population structure on the results, this study conducted weighted multivariable logistic regression analysis in the overall sample to evaluate factors associated with MHL attainment among residents after weighting. To test multicollinearity among independent variables in the multivariable models, the generalized variance inflation factor (GVIF) was used for assessment, and GVIF < 5 was considered to indicate no obvious multicollinearity (37). To further evaluate the urban–rural difference and its stability, stratified analyses were conducted after adjusting for gender, regional distribution, age, education level, occupation, marital status, income, smoking, alcohol use, SRH, and chronic disease status to assess differences in MHL attainment between rural and urban residents. Finally, Fairlie decomposition analysis was performed using Stata MP16.0 to explain the sources of urban–rural differences, and the model settings and related indicators are described in the following section. A *p-*value of <0.05 was considered statistically significant.

### Fairlie decomposition analysis

To analyze the contributions of various observable factors to the urban–rural difference in the MHL attainment rate and reduce the estimation bias that may be caused by the urban–rural composition of the sample, this study used a Fairlie nonlinear decomposition model based on urban–rural stratified weights to decompose the urban–rural difference. Because MHL attainment was a binary variable, when the Logistic model was used for estimation, the between-group difference was expressed as the difference in predicted probabilities. In this study, stratified weights for urban and rural residents were calculated according to the permanent resident population structure and the urban–rural composition of the survey sample, and the weights were incorporated into the Logistic model estimation and mean predicted probability calculation (38, 39).

Let the urban resident group be denoted as *U* and the rural resident group as *R*. ${\bar{Y}}_{w}^{U}$ and ${\bar{Y}}_{w}^{R}$ represent the weighted MHL attainment rates of urban and rural residents, respectively; $X^{U}$ and $X^{R}$ represent the matrices of explanatory variables for the two groups, respectively; $w_{i}^{U}$ and $w_{i}^{R}$ represent the stratified weights of urban and rural residents, respectively; F(·) denotes the Logistic cumulative distribution function; and ${\hat{\beta }}^{*}$ denotes the coefficient estimated from the weighted pooled-sample Logistic model. The urban–rural difference in the MHL attainment rate can then be expressed as:


$${\bar{Y}}_{w}^{U}-{\bar{Y}}_{w}^{R}=[\frac{\sum_{i=1}^{N^{U}}w_{i}^{U}F(X_{i}^{U}{\hat{\beta }}^{*})}{\sum_{i=1}^{N^{U}}w_{i}^{U}}-\frac{\sum_{i=1}^{N^{R}}w_{i}^{R}F(X_{i}^{R}{\hat{\beta }}^{*})}{\sum_{i=1}^{N^{R}}w_{i}^{R}}]+\varepsilon$$

where the term in brackets is the explained component, representing the difference in weighted mean predicted probabilities formed by differences in the distribution of observable characteristics between urban and rural residents; ε is the unexplained component, representing the difference caused by unobserved factors in the model and other factors not included in the model. To further calculate the contribution of each individual variable, the distributions of corresponding variables between urban and rural residents were sequentially replaced to estimate the change in the weighted mean predicted probability caused by that variable:


$$C_{k}=\frac{1}{\sum_{i=1}^{N}w_{i}}\sum_{i=1}^{N}w_{i}[F(X_{i,k}^{U}{\hat{\beta }}^{*}+X_{i,-k}^{R}{\hat{\beta }}^{*})-F(X_{i,k}^{R}{\hat{\beta }}^{*}+X_{i,-k}^{R}{\hat{\beta }}^{*})]$$

where Ck represents the absolute contribution of the kth variable to the urban–rural difference in the MHL attainment rate, and the contribution rate was calculated as $C_{k}/({\bar{Y}}_{w}^{U}-{\bar{Y}}_{w}^{R})\times 100\%$ (40, 41). In this study, the coefficients estimated from the weighted pooled-sample logistic regression model for urban and rural residents were used as reference coefficients to decompose the urban–rural difference in the MHL attainment rate. The contribution values and contribution rates of each factor were then calculated separately. In the sensitivity analysis, the decomposition was repeated after changing the reference coefficient. The impact of coefficient selection on the decomposition results was compared to verify the robustness of the decomposition results. Gender, regional distribution, age, education level, occupation, marital status, monthly personal income, monthly household income, alcohol use, smoking, SRH, and chronic disease status were included in the Fairlie decomposition model to estimate the contribution value, contribution rate, and statistical significance of each variable.

## Results

### Basic characteristics of the study participants

A total of 36,874 participants were included in this survey, including 18,950 males (51.39%) and 17,924 females (48.61%). In terms of urban–rural residence, 26,343 participants (71.44%) were from rural areas and 10,531 participants (28.56%) were from urban areas. The survey results showed that 67.78% of participants did not meet the standard for MHL (24,998/36,874), whereas 32.21% met the standard for MHL (11,876/36,874). After weighting according to the urban–rural population structure, the weighted attainment rate was 35.18%. The MHL attainment rate among urban residents was 37.08% (3,905/10,531), which was higher than that among rural residents at 30.26% (7,971/26,343) (*p* < 0.001). The results of univariate analysis showed that the distribution differences in all variables between urban and rural residents were statistically significant (Table 1).

**Table 1:** Demographic and urban–rural characteristics of the study participants.

Variables	Overall, *N* (%)	Rural, *N* (%)	Urban, *N* (%)	*χ* ^2^	*p*
Gender				424.796	<0.001
Male	18,950 (51.39)	14,432 (54.78)	4,518 (42.90)		
Female	17,924 (48.61)	11,911 (45.22)	6,013 (57.10)		
Region				7,785.083	<0.001
Urban districts	9,487 (25.73)	3,453 (13.11)	6,034 (57.30)		
New urban districts	12,644 (34.29)	10,428 (39.59)	2,216 (21.04)		
Northeast region	9,519 (25.81)	8,285 (31.45)	1,234 (11.72)		
Southeast region	5,224 (14.17)	4,177 (15.86)	1,047 (9.94)		
Age group (years)				1,273.467	<0.001
0–24	631 (1.71)	366 (1.39)	265 (2.52)		
25–39	5,925 (16.07)	3,254 (12.35)	2,671 (25.36)		
40–54	8,906 (24.15)	6,145 (23.33)	2,761 (26.22)		
55–64	10,602 (28.75)	8,049 (30.55)	2,553 (24.24)		
≥65	10,810 (29.32)	8,529 (32.38)	2,281 (21.66)		
Education level				4,665.922	<0.001
Illiterate or semi-literate	4,103 (11.13)	3,733 (14.17)	370 (3.51)		
Primary school	12,418 (33.68)	10,479 (39.78)	1939 (18.41)		
Junior middle school	9,930 (26.93)	7,104 (26.97)	2,826 (26.84)		
High school/vocational school	4,462 (12.10)	2,408 (9.14)	2054 (19.50)		
University or above	5,961 (16.17)	2,619 (9.94)	3,342 (31.73)		
Occupation				6,750.065	<0.001
Farmer/worker	21,763 (59.02)	18,954 (71.95)	2,809 (26.67)		
Government employee/technical professional	2,566 (6.96)	1,321 (5.01)	1,245 (11.82)		
Commercial/service worker	1747 (4.74)	828 (3.14)	919 (8.73)		
Retired	2,476 (6.71)	826 (3.14)	1,650 (15.67)		
Other	8,322 (22.57)	4,414 (16.76)	3,908 (37.11)		
Marital status				171.865	<0.001
Unmarried	2,503 (6.79)	1,503 (5.71)	1,000 (9.50)		
Married	30,764 (83.43)	22,207 (84.30)	8,557 (81.26)		
Divorced/widowed/other	3,607 (9.78)	2,633 (10.00)	974 (9.25)		
Monthly personal income (CNY)				3,884.514	<0.001
0–1,499	15,874 (43.05)	13,593 (51.60)	2,281 (21.66)		
1,500–2,999	10,433 (28.29)	7,385 (28.03)	3,048 (28.94)		
3,000–4,999	7,806 (21.17)	4,168 (15.82)	3,638 (34.55)		
5,000–7,999	2,179 (5.91)	930 (3.53)	1,249 (11.86)		
≥8,000	582 (1.58)	267 (1.01)	315 (2.99)		
Monthly household income (CNY)				2,457.536	<0.001
0–2,999	10,799 (29.29)	9,323 (35.39)	1,476 (14.02)		
3,000–5,999	11,579 (31.40)	8,519 (32.34)	3,060 (29.06)		
6,000–9,999	7,692 (20.86)	4,711 (17.88)	2,981 (28.31)		
10,000–19,999	4,280 (11.61)	2,270 (8.62)	2010 (19.09)		
≥20,000	2,524 (6.84)	1,520 (5.77)	1,004 (9.53)		
Alcohol use				131.607	<0.001
No	29,385 (79.69)	20,592 (78.17)	8,793 (83.50)		
Yes	7,489 (20.31)	5,751 (21.83)	1738 (16.50)		
Smoking				281.746	<0.001
No	27,750 (75.26)	19,196 (72.87)	8,554 (81.23)		
Yes	9,124 (24.74)	7,147 (27.13)	1977 (18.77)		
SRH				180.166	<0.001
Good	28,196 (76.47)	19,649 (74.59)	8,547 (81.16)		
Fair/poor	8,678 (23.53)	6,694 (25.41)	1984 (18.84)		
Chronic disease				235.871	<0.001
No	23,804 (64.55)	16,368 (62.13)	7,436 (70.61)		
Yes	13,070 (35.45)	9,975 (37.87)	3,095 (29.39)		

SRH, self-rated health.

### Characteristic distribution of MHL among rural and urban residents

The analysis of MHL attainment among rural and urban residents showed that the attainment rate increased with higher education level. Farmers or workers had a relatively lower attainment rate than participants in other occupations. Monthly personal income and monthly household income were associated with the attainment rate. Participants with alcohol use, smoking, fair or poor SRH, and chronic disease had lower attainment rates. The results of univariate analysis of the characteristic distributions in rural and urban populations showed that regional distribution, age group, education level, occupation, marital status, monthly personal income, monthly household income, alcohol use, smoking, SRH, and chronic disease status were statistically significant in relation to MHL attainment among rural and urban residents (Table 2).

**Table 2:** MHL proficiency among rural and urban residents by participant characteristics.

Variables	Overall	Rural				Urban			
	Adequate, *N* (%)	Inadequate, *N* (%)	Adequate, *N* (%)	*χ* ^2^	*p*	Inadequate, *N* (%)	Adequate, *N* (%)	*χ* ^2^	*p*
Gender				1.016	0.313			1.477	0.224
Male	5,974 (31.53)	10,103 (70.00)	4,329 (30.00)			2,873 (63.59)	1,645 (36.41)		
Female	5,902 (32.93)	8,269 (69.42)	3,642 (30.58)			3,753 (62.41)	2,260 (37.59)		
Region				290.723	<0.001			144.904	<0.001
Central urban area	2,888 (30.44)	2,704 (78.31)	749 (21.69)			3,895 (64.55)	2,139 (35.45)		
New urban area	4,388 (34.70)	6,743 (64.66)	3,685 (35.34)			1,513 (68.28)	703 (31.72)		
Northeastern Chongqing	2,758 (28.97)	6,048 (73.00)	2,237 (27.00)			713 (57.78)	521 (42.22)		
Southeastern Chongqing	1842 (35.26)	2,877 (68.88)	1,300 (31.12)			505 (48.23)	542 (51.77)		
Age group (years)				30.663	<0.001			12.501	0.014
0–24	193 (30.59)	274 (74.86)	92 (25.14)			164 (61.89)	101 (38.11)		
25–39	2054 (34.67)	2,231 (68.56)	1,023 (31.44)			1,640 (61.40)	1,031 (38.60)		
40–54	3,007 (33.76)	4,198 (68.32)	1947 (31.68)			1701 (61.61)	1,060 (38.39)		
55–64	3,426 (32.31)	5,552 (68.98)	2,497 (31.02)			1,624 (63.61)	929 (36.39)		
≥65	3,196 (29.57)	6,117 (71.72)	2,412 (28.28)			1,497 (65.63)	784 (34.37)		
Education level				75.392	<0.001			19.132	0.001
Illiterate or semi-literate	1,057 (25.76)	2,791 (74.77)	942 (25.23)			255 (68.92)	115 (31.08)		
Primary school	3,892 (31.34)	7,253 (69.21)	3,226 (30.79)			1,273 (65.65)	666 (34.35)		
Junior middle school	3,157 (31.79)	4,984 (70.16)	2,120 (29.84)			1789 (63.31)	1,037 (36.69)		
High school/vocational school	1,615 (36.19)	1,569 (65.16)	839 (34.84)			1,278 (62.22)	776 (37.78)		
University or above	2,155 (36.15)	1775 (67.77)	844 (32.23)			2031 (60.77)	1,311 (39.23)		
Occupation				31.964	<0.001			18.822	0.001
Farmer/worker	6,536 (30.03)	13,370 (70.54)	5,584 (29.46)			1857 (66.11)	952 (33.89)		
Government employee/technical professional	929 (36.20)	850 (64.35)	471 (35.65)			787 (63.21)	458 (36.79)		
Commercial/service worker	624 (35.72)	553 (66.79)	275 (33.21)			570 (62.02)	349 (37.98)		
Retired	899 (36.31)	551 (66.71)	275 (33.29)			1,026 (62.18)	624 (37.82)		
Other	2,888 (34.70)	3,048 (69.05)	1,366 (30.95)			2,386 (61.05)	1,522 (38.95)		
Marital status				4.473	0.107			15.088	0.001
Unmarried	865 (34.56)	1,051 (69.93)	452 (30.07)			587 (58.70)	413 (41.30)		
Married	9,941 (32.31)	15,438 (69.52)	6,769 (30.48)			5,385 (62.93)	3,172 (37.07)		
Divorced/widowed/other	1,070 (29.66)	1883 (71.52)	750 (28.48)			654 (67.15)	320 (32.85)		
Monthly personal income (CNY)				22.132	<0.001			15.479	0.004
0–1,499	4,748 (29.91)	9,649 (70.99)	3,944 (29.01)			1,477 (64.75)	804 (35.25)		
1,500–2,999	3,420 (32.78)	5,062 (68.54)	2,323 (31.46)			1951 (64.01)	1,097 (35.99)		
3,000–4,999	2,736 (35.05)	2,848 (68.33)	1,320 (31.67)			2,222 (61.08)	1,416 (38.92)		
5,000–7,999	793 (36.39)	624 (67.10)	306 (32.90)			762 (61.01)	487 (38.99)		
≥8,000	179 (30.76)	189 (70.79)	78 (29.21)			214 (67.94)	101 (32.06)		
Monthly household income (CNY)				59.348	<0.001			24.070	<0.001
0–2,999	3,066 (28.39)	6,756 (72.47)	2,567 (27.53)			977 (66.19)	499 (33.81)		
3,000–5,999	3,734 (32.25)	5,862 (68.81)	2,657 (31.19)			1983 (64.80)	1,077 (35.20)		
6,000–9,999	2,692 (35.00)	3,137 (66.59)	1,574 (33.41)			1863 (62.50)	1,118 (37.50)		
10,000–19,999	1,508 (35.23)	1,569 (69.12)	701 (30.88)			1,203 (59.85)	807 (40.15)		
≥20,000	876 (34.71)	1,048 (68.95)	472 (31.05)			600 (59.76)	404 (40.24)		
Alcohol use				3.880	0.049			2.310	0.129
No	9,581 (32.61)	14,300 (69.44)	6,292 (30.56)			5,504 (62.60)	3,289 (37.40)		
Yes	2,295 (30.64)	4,072 (70.81)	1,679 (29.19)			1,122 (64.56)	616 (35.44)		
Smoking				22.165	<0.001			5.548	0.019
No	9,183 (33.09)	13,231 (68.93)	5,965 (31.07)			5,336 (62.38)	3,218 (37.62)		
Yes	2,693 (29.52)	5,141 (71.93)	2006 (28.07)			1,290 (65.25)	687 (34.75)		
SRH				149.586	<0.001			125.559	<0.001
Good	9,730 (34.51)	13,306 (67.72)	6,343 (32.28)			5,160 (60.37)	3,387 (39.63)		
Fair/poor	2,146 (24.73)	5,066 (75.68)	1,628 (24.32)			1,466 (73.89)	518 (26.11)		
Chronic disease				19.277	<0.001			95.061	<0.001
No	8,090 (33.99)	11,256 (68.77)	5,112 (31.23)			4,458 (59.95)	2,978 (40.05)		
Yes	3,786 (28.97)	7,116 (71.34)	2,859 (28.66)			2,168 (70.05)	927 (29.95)		

SRH, self-rated health; MHL, mental health literacy.

### Logistic regression analysis of MHL proficiency among rural and urban residents

**Figure 1:**
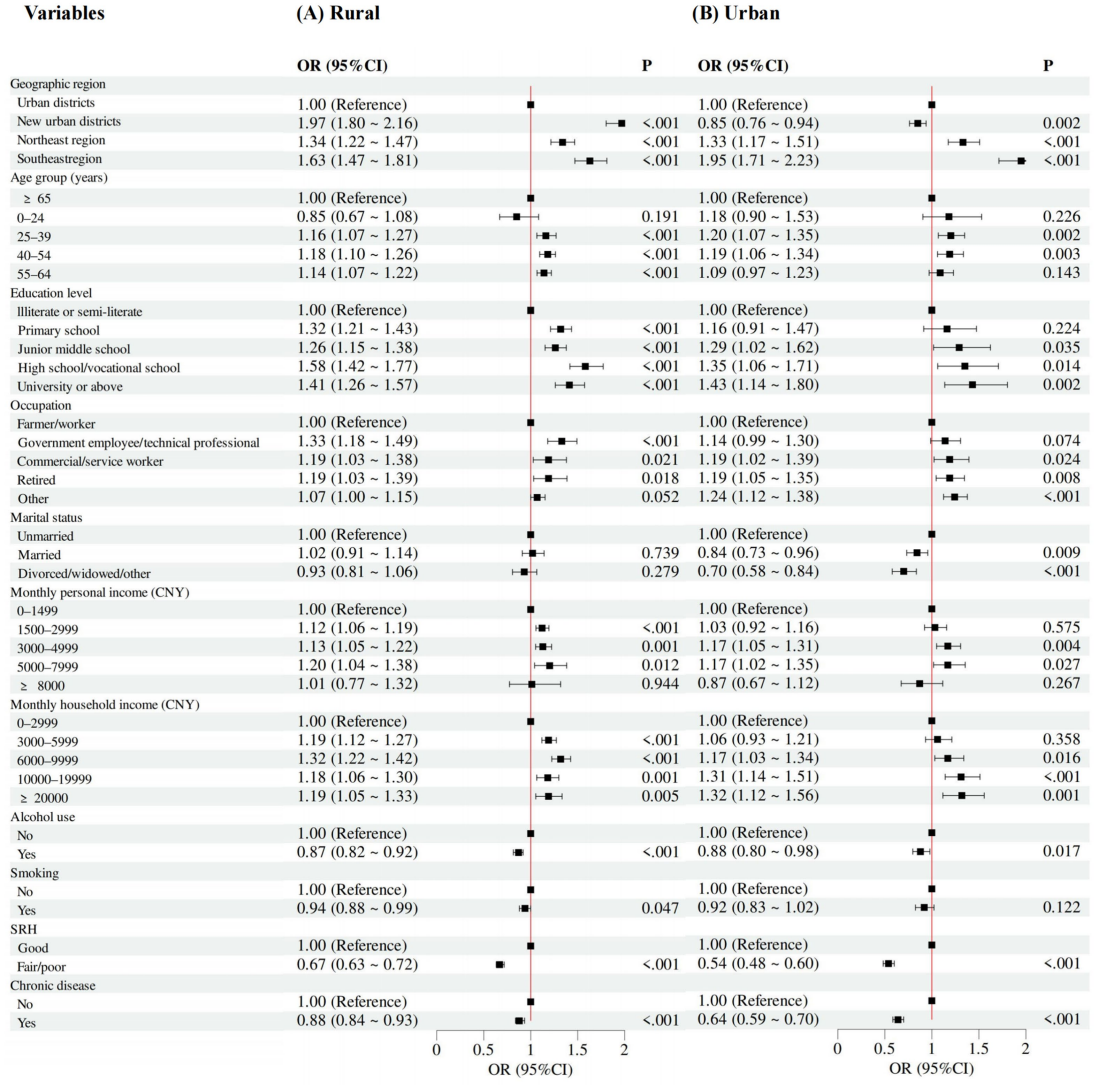
Forest plot of multivariable logistic regression for factors associated with MHL proficiency among rural and urban residents. SRH, self-rated health; MHL, mental health literacy.

Figure 1 and Supplementary Table 1 show the results of the logistic regression models for meeting the standard for MHL among rural and urban residents. Among rural residents, compared with the reference group, residents in different regional distributions (new urban districts, OR = 1.97; Northeast region, OR = 1.34; southeast region, OR = 1.63), age groups (25–39 years, OR = 1.16; 40–54 years, OR = 1.18; 55–64 years, OR = 1.14), education levels (primary school, OR = 1.32; junior middle school, OR = 1.26; high school/vocational school, OR = 1.58; university or above, OR = 1.41), occupations (government employee/technical professional, OR = 1.33; commercial/service worker, OR = 1.19; retired, OR = 1.19), monthly personal income groups (1,500–2,999 CNY, OR = 1.12; 3,000–4,999 CNY, OR = 1.13; 5,000–7,999 CNY, OR = 1.20), and monthly household income groups (3,000–5,999 CNY, OR = 1.19; 6,000–9,999 CNY, OR = 1.32; 10,000–19,999 CNY, OR = 1.18; ≥20,000 CNY, OR = 1.19) had higher rates of meeting the standard for MHL. Residents who smoked (OR = 0.94), used alcohol (OR = 0.87), had fair or poor SRH (OR = 0.67), or had chronic disease (OR = 0.88) had lower rates of meeting the standard for MHL.

Among urban residents, compared with the reference group, residents in different regional distributions (new urban districts, OR = 0.85; northeast region, OR = 1.33; southeast region, OR = 1.95), age groups (25–39 years, OR = 1.20; 40–54 years, OR = 1.19), education levels (junior middle school, OR = 1.29; high school/vocational school, OR = 1.35; university or above, OR = 1.43), occupations (commercial/service worker, OR = 1.19; retired, OR = 1.19; other, OR = 1.24), monthly personal income groups (3,000–4,999 CNY, OR = 1.17; 5,000–7,999 CNY, OR = 1.17), and monthly household income groups (6,000–9,999 CNY, OR = 1.17; 10,000–19,999 CNY, OR = 1.31; ≥20,000 CNY, OR = 1.32) had higher rates of meeting the standard for MHL. Married residents (OR = 0.84), divorced/widowed/other residents (OR = 0.70), residents who smoked (OR = 0.92), those with fair or poor SRH (OR = 0.54), and those with chronic disease (OR = 0.64) had lower rates of meeting the standard for MHL.

. Overall, the results among rural and urban residents showed that MHL attainment was associated with regional distribution, age, education level, occupation, income level, marital status, alcohol use, smoking, SRH, and chronic disease status.

### Logistic regression analysis of MHL proficiency after urban–rural weighting

The results of the analysis of factors associated with residents’ MHL after urban–rural weighting showed that urban residents had higher odds of meeting the standard for MHL than rural residents (OR = 1.36, 95%CI: 1.29–1.43). The results for other variables were generally consistent with the unweighted results, and the associations of MHL attainment with age, education level, occupation, income level, marital status, alcohol use, smoking, SRH, and chronic disease status were relatively robust. However, after weighting, except for the Southeast region, the associations between other regions and MHL attainment were no longer statistically significant (Table 3).

**Table 3:** Logistic regression results for factors associated with MHL proficiency among residents after urban–rural weighting.

Variables	*β*	S $\bar{x}$	Wald *χ*^2^	*p*	OR (95%CI)
Urban–rural residence					
Rural					1.00 (Reference)
Urban	0.31	0.02	150.71	<0.001	1.36 (1.29–1.43)
Region					
Urban districts					1.00 (Reference)
New urban districts	−0.05	0.03	3.22	0.073	0.95 (0.90–1.00)
Northeast region	0.01	0.03	0.04	0.840	1.01 (0.95–1.07)
Southeast region	0.40	0.03	133.54	<0.001	1.50 (1.40–1.60)
Age group (years)					
≥65					1.00 (Reference)
0–24	0.16	0.08	4.63	0.032	1.18 (1.01–1.37)
25–39	0.23	0.03	53.25	<0.001	1.26 (1.19–1.35)
40–54	0.20	0.03	41.97	<0.001	1.22 (1.15–1.30)
55–64	0.11	0.03	13.00	<0.001	1.12 (1.05–1.19)
Education level					
Illiterate or semi-literate					1.00 (Reference)
Primary school	0.25	0.05	23.60	<0.001	1.28 (1.16–1.42)
Junior middle school	0.34	0.05	45.37	<0.001	1.40 (1.27–1.55)
High school/vocational school	0.45	0.05	72.54	<0.001	1.57 (1.41–1.74)
University or above	0.50	0.05	97.82	<0.001	1.65 (1.49–1.82)
Occupation					
Farmer/worker					1.00 (Reference)
Government employee/technical professional	0.22	0.04	33.11	<0.001	1.25 (1.16–1.35)
Commercial/service worker	0.26	0.04	33.73	<0.001	1.29 (1.18–1.41)
Retired	0.26	0.04	53.12	<0.001	1.30 (1.21–1.39)
Other	0.27	0.03	106.99	<0.001	1.31 (1.25–1.38)
Marital status					
Unmarried					1.00 (Reference)
Married	−0.17	0.04	19.68	<0.001	0.84 (0.78–0.91)
Divorced/widowed/other	−0.33	0.05	41.74	<0.001	0.72 (0.65–0.79)
Monthly personal income (CNY)					
0–1,499					1.00 (Reference)
1,500–2,999	0.11	0.03	15.11	<0.001	1.12 (1.06–1.18)
3,000–4,999	0.25	0.03	74.48	<0.001	1.28 (1.21–1.35)
5,000–7,999	0.27	0.04	44.33	<0.001	1.31 (1.21–1.41)
≥8,000	−0.02	0.07	0.11	0.745	0.98 (0.84–1.13)
Monthly household income (CNY)					
0–2,999					1.00 (Reference)
3,000–5,999	0.15	0.03	21.61	<0.001	1.16 (1.09–1.24)
6,000–9,999	0.27	0.03	65.75	<0.001	1.31 (1.23–1.40)
10,000–19,999	0.36	0.04	94.70	<0.001	1.43 (1.33–1.54)
≥20,000	0.35	0.04	59.96	<0.001	1.41 (1.29–1.54)
Alcohol use					
No					1.00 (Reference)
Yes	−0.10	0.03	12.13	<0.001	0.90 (0.86–0.96)
Smoking					
No					1.00 (Reference)
Yes	−0.16	0.03	34.45	<0.001	0.85 (0.81–0.90)
SRH					
Good					1.00 (Reference)
Fair/poor	−0.57	0.03	388.76	<0.001	0.57 (0.53–0.60)
Chronic disease					
No					1.00 (Reference)
Yes	−0.37	0.02	239.56	<0.001	0.69 (0.66–0.72)

SRH, self-rated health; MHL, mental health literacy.

### Comparative analysis of MHL proficiency among rural and urban residents

**Figure 2:**
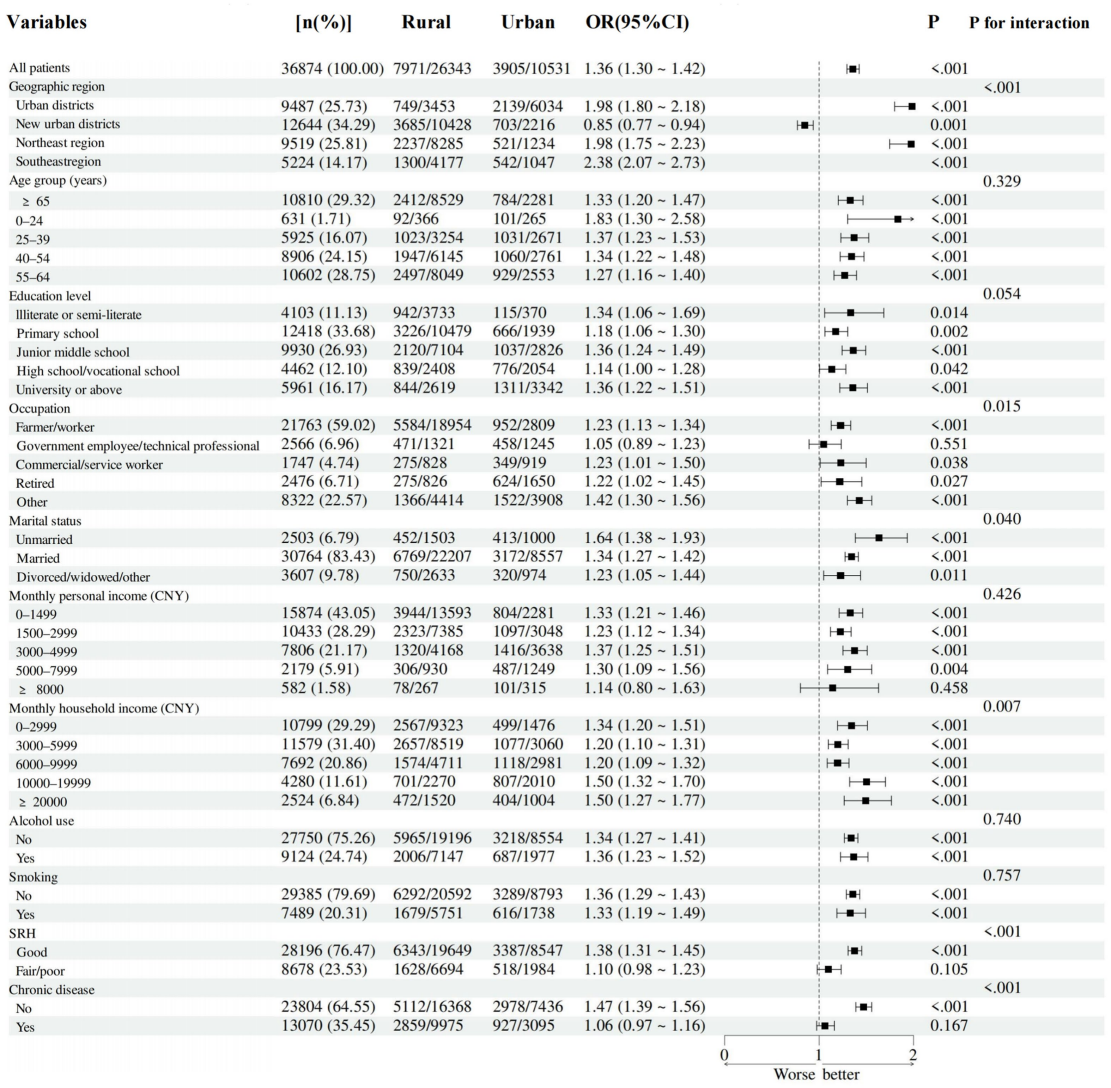
Forest plot of stratified analysis of MHL proficiency among rural and urban residents. Adjusted for gender, regional distribution, age, education level, occupation, marital status, income, smoking, alcohol use, SRH, and chronic disease status. SRH, self-rated health; MHL, mental health literacy.

To further examine the difference in MHL attainment between urban and rural residents and the robustness of urban–rural differences across different variable strata, we conducted stratified analyses after adjusting for statistically significant covariates (Figure 2). The results showed that, in the overall study population, urban residents had a higher level of MHL than rural residents (OR = 1.36, 95%CI: 1.30–1.42). Across variable strata, except for a small number of subgroups such as new urban districts, government employee/technical professional, monthly personal income of ≥8,000 CNY, fair or poor SRH, and chronic disease, where the results were inconsistent or not statistically significant, urban residents showed higher MHL attainment than rural residents in most variable strata, indicating that the urban–rural difference in MHL attainment was relatively significant and stable.

### Decomposition analysis results

Using the pooled coefficients as the reference coefficients in the weighted Fairlie nonlinear decomposition model (Table 4), the results showed that the urban–rural difference in the MHL attainment rate was 0.0682. Of this difference, 0.0240 (35.17%) could be explained by the observable covariates included in the model, whereas 0.0442 (64.83%) could not be explained by the included observable covariates, suggesting that unobserved factors or differences may still exist between urban and rural areas in addition to the variables included in the model. Specifically, regional distribution (−35.13%, *β* = −0.024, *p* < 0.001), age (−9.41%, *β* = −0.006, *p* < 0.001), occupation (27.01%, *β* = 0.018, *p* < 0.001), education level (26.55%, *β* = 0.018, *p* < 0.001), monthly household income (12.90%, *β* = 0.009, *p* < 0.001), monthly personal income (−6.97%, *β* = −0.005, *p* < 0.001), SRH (7.90%, *β* = 0.005, *p* < 0.001), smoking (3.41%, *β* = 0.002, *p* < 0.001), chronic disease status (5.39%, *β* = 0.004, *p* < 0.001), marital status (2.66%, *β* = 0.002, *p* < 0.001), alcohol use (0.38%, *β* < 0.001, *p* < 0.001), and gender (0.48%, *β* < 0.001, *p* < 0.001) all made statistically significant contributions to the urban–rural difference in the MHL attainment rate. Overall, the urban–rural difference in the MHL attainment rate was mainly associated with factors such as regional distribution, age structure, occupational structure, education level, household economic conditions, and SRH. This study further conducted sensitivity analyses using the urban-sample coefficients and rural-sample coefficients separately for decomposition. The results showed some changes after using the urban or rural sample coefficients compared with the pooled-sample coefficients, but the contribution directions and magnitudes of the relevant factors were generally stable (Supplementary Tables 2, 3).

**Table 4:** Weighted Fairlie decomposition results for urban–rural differences in MHL proficiency.

Terms of decomposition	Mental health literacy proficiency
Difference	0.0682249
Explained (%)	0.0239949 (35.17%)
Non-explained (%)	0.0442300 (64.83%)
Explained	

Contribution to difference	*p*	*β*	Contribution (%)	[95%CI]
Gender	<0.001	0.0003243	0.48	(0.0002897, 0.0003589)
Region	<0.001	−0.0239705	−35.13	(−0.0248627, −0.0230782)
Age group (years)	<0.001	−0.0064222	−9.41	(−0.0069658, −0.0058785)
Education level	<0.001	0.0181156	26.55	(0.0175207, 0.0187105)
Occupation	<0.001	0.0184303	27.01	(0.0179062, 0.0189544)
Marital status	<0.001	0.0018160	2.66	(0.0014768, 0.0021551)
Monthly personal income (CNY)	<0.001	−0.0047525	−6.97	(−0.0050648, −0.0044402)
Monthly household income (CNY)	<0.001	0.0087995	12.90	(0.0083819, 0.0092171)
Smoking	<0.001	0.0023246	3.41	(0.0020354, 0.0026138)
Alcohol use	<0.001	0.0002622	0.38	(0.0002177, 0.0003066)
SRH	<0.001	0.0053912	7.90	(0.0047406, 0.0060419)
Chronic disease	<0.001	0.0036764	5.39	(0.0031390, 0.0042137)

SRH, self-rated health; MHL, mental health literacy.

## Discussion

Based on survey data from 36,874 community residents in Chongqing, this study analyzed the level of MHL attainment, related influencing factors, and sources of urban–rural differences among residents in Southwest China. Unlike previous studies that mostly focused on a single region, specific age group, or certain type of mental health outcome, this study included both urban and rural residents across the entire municipality and combined the weighted MHL attainment rate based on the urban–rural population structure, multivariable logistic regression, and the weighted Fairlie nonlinear decomposition model to comprehensively evaluate individual factors associated with MHL attainment and the contributions to urban–rural differences. MHL not only involves mastery of mental health knowledge, but also reflects residents’ ability to identify, cope with, and seek help for mental health problems. In recent years, research on digital MHL and the promotion of MHL in schools and workplaces has gradually increased, suggesting that MHL has expanded from general knowledge popularization to a comprehensive public health indicator covering prevention, identification, and service utilization (42–44). The present study analyzed MHL at the level of general residents and may provide population-based evidence for optimizing mental health promotion and primary mental health service strategies in Southwest China.

This study showed that the overall MHL attainment rate among Chongqing residents was 32.21%, with 37.08% among urban residents and 30.26% among rural residents, indicating a higher rate among urban residents than among rural residents. After weighting according to the urban–rural population structure, the overall attainment rate was 35.18%, which was higher than the unweighted result, suggesting that because the proportion of rural residents in this study sample was relatively high, the unweighted estimate may have underestimated the overall level of MHL among Chongqing residents to some extent. However, the direction of the urban–rural difference did not change after weighting, and urban residents still had higher MHL than rural residents. Regarding attainment rates within urban areas, the MHL attainment rate in southeastern Chongqing was higher than that in the central urban area. The sample size of urban residents in the central urban area was 6,034, whereas that in southeastern Chongqing was 1,047, and the difference between the two regions may have been caused by differences in sample size and sample structure. Meanwhile, we considered relevant aspects such as mental health promotion policies, financial investment, and the implementation of related publicity and promotion activities in the two regions, and did not identify significant differences in mental health promotion policies between them (25, 45). Therefore, sample size and sample realization differences may be the main reasons for the difference in attainment rates between the two regions. Compared with relevant policy targets and survey results, the findings of this survey were higher than the phased targets proposed in the Healthy China Action (2019–2030), which required the level of MHL among residents to reach 20% by 2022 and 30% by 2030 (17), and were also higher than the requirement in the Three-Year Action Plan for the Construction of the Mental Health System in Chongqing (2023–2025), which proposed that the level of MHL among residents should be no lower than 30% by 2025 (25). However, it should be noted that this study was a cross-sectional questionnaire survey and may have been affected by social desirability, a tendency toward positive responses, and the survey organization method; therefore, the attainment rate may have been overestimated to some extent. In addition, the attainment rate in this study was higher than the MHL attainment rate reported by Li et al. (46) in a survey of community residents during the re-outbreak stage of the COVID-19 epidemic in China, and was also higher than the overall MHL level reported in a survey of residents in Foshan (47). However, these differences may also be related to differences in survey regions, survey periods, sample structures, and the extent to which mental health promotion work had been advanced. Huang et al. (48) based on a sample of Chinese college students, found that MHL moderated the relationship between bullying victimization and anxiety and depressive symptoms, suggesting that higher MHL may weaken the risk of negative life experiences being transformed into psychological symptoms. A chain mediation model of attitudes toward professional psychological help-seeking among Chinese residents by Yang et al. further showed that MHL may influence attitudes toward professional psychological help-seeking through distress disclosure and social support (49). Yao et al. (50) further showed in a study of 5,578 Chinese college students that MHL was negatively associated with depression, anxiety, and suicidal ideation. These studies suggest that MHL is not only a basic capacity for residents to acquire mental health knowledge and identify psychological problems, but may also reduce the risk of mental health problems through pathways such as promoting emotional expression, improving the utilization of social support, and enhancing attitudes toward professional help-seeking. Meanwhile, compared with the above studies, the present study covered a broader sample and used urban–rural differences as the core analytical dimension, suggesting that improvements in MHL should continue to take regional differences and population equity into account.

After weighted logistic regression based on the urban–rural population structure, the directions of association for variables such as age, education level, occupation, income level, marital status, smoking, alcohol use, SRH, and chronic disease status were generally consistent with the unweighted results. The results showed that, compared with the group aged ≥65 years, rural residents aged 25–39, 40–54, and 55–64 years had higher odds of meeting the standard for MHL, suggesting that young and middle-aged adults and middle-aged and older adults may have stronger abilities to access information, understand knowledge, and receive health communication. A survey of MHL among Shanghai residents also found that younger age and higher education level were associated with better MHL (51). Education level was positively associated with MHL, which is consistent with domestic and international studies on influencing factors of MHL and general health literacy (46, 51), indicating that higher education level may help enhance the ability to identify mental health problems, screen health information, and actively seek help. In terms of occupation, among urban residents, commercial/service workers, retired individuals, and those in other occupations had higher attainment rates than farmers or workers, which may be related to differences in the scope of social interaction, opportunities for exposure to health information, and service accessibility. In terms of income, residents with higher personal and household income had relatively higher attainment rates, and the association between low income and limited health literacy has also been consistently reported in systematic reviews (52). In addition, this study observed that, in the urban sample, unmarried participants had a higher MHL attainment rate than married participants and those who were divorced, widowed, or in other marital status groups. This finding is consistent with some previous surveys. This result may be related to the younger age structure and higher education level of unmarried urban populations, as well as their greater opportunities to access mental health information through school education and internet platforms (53, 54). However, in the study sample, married participants accounted for 83.43%, whereas unmarried participants and those who were divorced, widowed, or in other marital status groups accounted for relatively low proportions, which may also be related to sample bias.

In terms of health behaviors, smokers and alcohol users had lower MHL attainment rates, which is similar to the findings of a meta-analysis showing that individuals with insufficient health literacy were more likely to engage in risk behaviors such as smoking (55). In addition, participants with fair or poor SRH and those with chronic disease had lower attainment rates, which is consistent with findings that insufficient health literacy may affect health information comprehension, long-term disease management, and health service utilization (56–59). A meta-analysis by Shao et al. (59) on health literacy interventions among patients with chronic diseases showed that health literacy interventions were helpful in improving related outcomes among patients with chronic diseases, which also suggests, from an intervention perspective, the necessity of integrating chronic disease management with mental health education. In terms of urban–rural residence, both the urban–rural weighted logistic regression and subgroup analyses showed that urban residents had higher MHL than rural residents, which is consistent with studies on MHL among urban and rural residents in Guangdong Province and studies on urban–rural differences in eHealth literacy (15, 24). In terms of regional distribution, after weighting based on the urban–rural population structure, the differences between new urban districts and urban districts, and between the Northeast region and urban districts, were no longer statistically significant, suggesting that some regional differences in the unweighted analysis may have been affected by the urban–rural sample composition and regional population structure. The Southeast region remained higher than urban districts after weighting, which may be related to the relatively small sample size in this region and to the fact that, due to mountainous terrain and transportation constraints, the actual survey may have tended to include survey sites with higher levels of resident concentration and stronger primary-level organizational mobilization capacity. Therefore, the surveyed population may have had some non-sampling error or sample realization bias (60), and this finding cannot be attributed to advantages in regional socioeconomic development level or mental health services.

The weighted Fairlie decomposition results showed that the urban–rural difference in the MHL attainment rate was 0.0682, of which 35.17% could be explained by the observable factors included in the model, whereas 64.83% came from the unexplained component. In addition to the pooled coefficients, this study also repeated the Fairlie decomposition using the urban sample coefficients and rural sample coefficients as sensitivity analyses. The results showed that the decomposition effects and related factors were relatively stable. Among the explained factors, occupation, education level, and monthly household income made relatively large positive contributions to the urban–rural difference, suggesting that socioeconomic factors are important sources of urban–rural disparities in MHL. A decomposition study by Tuersun et al. (23) on urban–rural differences in anxiety among Chinese college students showed that household income, exercise habits, and psychological symptoms made important contributions to the differences. The Fairlie decomposition of urban–rural differences in eHealth literacy among Chinese college students by Yu et al. (24) also found that 28.6% of the difference was attributable to observed factors, with household income, exercise habits, and depressive symptoms being the main contributing factors. In addition, a Fairlie decomposition study by Xie et al. (61) on urban–rural differences in depressive symptoms among employed populations in China showed that education level, SRH, and chronic disease made important contributions to urban–rural differences in depressive symptoms, with education level contributing 39.10%. Fu et al. (62), based on data from the China Family Panel Studies, also found that the education level of the household head, SRH, and economic-related factors had explanatory effects on urban–rural differences in catastrophic health expenditure among households with patients with chronic non-communicable diseases. However, 64.83% of the difference in this study still could not be explained by the existing covariates, indicating that, in addition to demographic, socioeconomic, and health status factors, urban–rural differences in MHL may also be affected by unmeasured factors such as accessibility of primary mental health services, exposure to health education, access to digital information, community support, mental health stigma, and utilization of mental health services. Regional distribution showed a negative contribution of a certain proportion, suggesting that, under the current sample and weighted structure, the internal regional distribution of Chongqing did not widen the urban–rural difference, but may instead have partially offset the urban–rural gap. This may be related to the fact that new urban districts, the Northeast region, and the Southeast region all include both urban and rural populations, and the differences in urban–rural attainment rates within different regions were not completely consistent. It may also reflect a possible shift in which some rural samples came from survey sites with higher resident concentration or better survey mobilization. Meanwhile, floating populations and migrant workers currently living in urban areas may not necessarily have higher MHL than rural residents in other regions because of their high mobility and weak sense of community belonging. The negative contribution of regional distribution may therefore be the result of the interplay of these factors.

Based on the findings of this survey, we propose the following public health recommendations for improving residents’ MHL.

First, the attainment rate was higher among urban residents than among rural residents, suggesting that rural areas may still have limitations in access to health information, coverage of mental health education, and service accessibility. Future efforts should strengthen the popularization of mental health knowledge in rural areas and promote equitable improvement in MHL between urban and rural populations.

Second, individuals with low education levels, farmers or workers, low-income populations, and those with poorer health status were less likely to meet the standard for MHL. It is recommended that MHL education be integrated into chronic disease follow-up and community health education, with particular attention paid to community health education for these populations.

Third, different regions of Chongqing include urban areas, rural areas, urban–rural fringe areas, and floating populations. Urban areas may also include groups with relatively low MHL. Therefore, interventions should be designed and implemented according to the actual conditions of the permanent resident population.

Fourth, the coordinated improvement of digital health literacy and MHL should be promoted. Internet platforms and short videos have become important information channels. It is recommended that mental health popularization be combined with digital health education, while improving the accessibility of digital approaches for rural residents, older adults, and populations with low education levels as much as possible.

### Strengths and limitations

This study has the following strengths. First, it adopted a citywide multistage random sampling design, with a large sample size covering different regional divisions and both urban and rural residents in Chongqing, which enabled a relatively comprehensive reflection of the MHL status of residents in Chongqing, Southwest China. Second, this study used the National Mental Health Literacy Questionnaire to assess MHL attainment, which has good public health interpretability, and incorporated demographic, socioeconomic, health behavior, and health status variables. Third, in addition to using logistic regression to identify influencing factors, this study further applied a weighted Fairlie nonlinear decomposition model and repeated the Fairlie decomposition after adjusting the sample coefficients as a sensitivity analysis to quantify the sources of urban–rural differences. This relatively adjusted the urban–rural sample structure, promoted the stability of the results, and improved the interpretive value of the findings.

This study also has several limitations. First, the cross-sectional design cannot support causal inference, and MHL may have bidirectional associations with related demographic and socioeconomic factors and health behaviors. Second, the questionnaire data mainly relied on self-report, which may be subject to recall bias and social desirability bias; therefore, the survey results are more suitable as phased monitoring and data reference. Third, although this study conducted weighted analysis based on the urban–rural population structure, the sampling design was not proportionally allocated according to urban–rural residence or other demographic and socioeconomic characteristics. Therefore, weighting could not be performed when separately analyzing attainment rates within urban and rural populations, and structural deviations within urban and rural groups may still exist. The potential differences between self-reported urban–rural categories and household registration categories also need to be further explored. At the same time, the response status in the Southeast region may have been affected by sample size, transportation accessibility, and the sample survey process. Fourth, although this study included demographic, socioeconomic, and health-related variables, it did not include variables such as history of mental illness, accessibility of mental health services, internet use, social support, mental health stigma, and sources of health information. Future studies may combine longitudinal follow-up and intervention research to further identify the sources of differences in MHL and the key pathways for its improvement.

## Conclusion

In summary, this study found that the overall MHL attainment rate among Chongqing residents was at a moderately high level but could still be further improved, and that urban residents had a higher MHL attainment rate than rural residents. Age, education level, occupation, income level, smoking, alcohol use, SRH, and chronic disease status were associated with MHL attainment. The Fairlie decomposition showed that urban–rural differences were partly explained by factors such as regional distribution, age, occupation, education level, household income, and SRH, among which socioeconomic factors made relatively prominent contributions. In the future, on the basis of continuing to promote mental health education for the whole population, greater attention should be paid to rural residents, individuals with low education levels, farmers or workers, low-income populations, and those with poorer health status. By integrating primary healthcare services, community health education, and digital health communication, residents’ mental health knowledge, disease recognition, and active help-seeking abilities can be improved, thereby promoting the equitable improvement of MHL between urban and rural populations.

## Data Availability

The original contributions presented in the study are included in the article/Supplementary material, further inquiries can be directed to the corresponding authors.
