# Supplementary material for: Analysis of influencing factors and urban–rural difference decomposition of mental health literacy among residents in Chongqing, Southwest China: a large-scale multistage random sampling survey
Source: Front Public Health. 2026 Aug 5;14:1895974. doi: 10.3389/fpubh.2026.1895974 (PMC13485747; doi:10.3389/fpubh.2026.1895974)
Supplement: Supplementary file 1 [file Table_1.DOCX]

***Supplementary Material***

**Supplementary table 1.** Logistic regression results for factors associated with MHL proficiency among rural and urban residents

| **Variables** | **Rural** | | | | | **Urban** | | | | |
| --- | --- | --- | --- | --- | --- | --- | --- | --- | --- | --- |
|  | ***β*** | ***S***$\bar{\text{x}}$ | ***Wald χ2*** | ***P*** | ***OR（95%CI）*** | ***β*** | ***S***$\bar{\text{x}}$ | ***Wald χ2*** | ***P*** | ***OR（95%CI）*** |
| **Region** |  |  |  |  |  |  |  |  |  |  |
| Urban districts |  |  |  |  | 1.00 (Reference) |  |  |  |  | 1.00 (Reference) |
| New urban districts | 0.68 | 0.05 | 217.27 | <.001 | 1.97 (1.80 ~ 2.16) | -0.17 | 0.05 | 9.92 | 0.002 | 0.85 (0.76 ~ 0.94) |
| Northeast region | 0.29 | 0.05 | 36.12 | <.001 | 1.34 (1.22 ~ 1.47) | 0.29 | 0.06 | 20.16 | <.001 | 1.33 (1.17 ~ 1.51) |
| Southeast region | 0.49 | 0.05 | 84.82 | <.001 | 1.63 (1.47 ~ 1.81) | 0.67 | 0.07 | 98.60 | <.001 | 1.95 (1.71 ~ 2.23) |
| **Age group (years)** |  |  |  |  |  |  |  |  |  |  |
| ≥65 |  |  |  |  | 1.00 (Reference) |  |  |  |  | 1.00 (Reference) |
| 0–24 | -0.16 | 0.12 | 1.72 | 0.191 | 0.85 (0.67 ~ 1.08) | 0.16 | 0.13 | 1.46 | 0.226 | 1.18 (0.90 ~ 1.53) |
| 25–39 | 0.15 | 0.04 | 11.36 | <.001 | 1.16 (1.07 ~ 1.27) | 0.18 | 0.06 | 9.49 | 0.002 | 1.20 (1.07 ~ 1.35) |
| 40–54 | 0.16 | 0.04 | 19.80 | <.001 | 1.18 (1.10 ~ 1.26) | 0.17 | 0.06 | 8.70 | 0.003 | 1.19 (1.06 ~ 1.34) |
| 55–64 | 0.13 | 0.03 | 14.90 | <.001 | 1.14 (1.07 ~ 1.22) | 0.09 | 0.06 | 2.13 | 0.143 | 1.09 (0.97 ~ 1.23) |
| **Education level** |  |  |  |  |  |  |  |  |  |  |
| Illiterate or semi-literate |  |  |  |  | 1.00 (Reference) |  |  |  |  | 1.00 (Reference) |
| Primary school | 0.28 | 0.04 | 40.83 | <.001 | 1.32 (1.21 ~ 1.43) | 0.15 | 0.12 | 1.49 | 0.224 | 1.16 (0.91 ~ 1.47) |
| Junior middle school | 0.23 | 0.05 | 25.60 | <.001 | 1.26 (1.15 ~ 1.38) | 0.25 | 0.12 | 4.45 | 0.035 | 1.29 (1.02 ~ 1.62) |
| High school/vocational school | 0.46 | 0.06 | 65.12 | <.001 | 1.58 (1.42 ~ 1.77) | 0.30 | 0.12 | 6.00 | 0.014 | 1.35 (1.06 ~ 1.71) |
| University or above | 0.34 | 0.06 | 37.09 | <.001 | 1.41 (1.26 ~ 1.57) | 0.36 | 0.12 | 9.24 | 0.002 | 1.43 (1.14 ~ 1.80) |
| **Occupation** |  |  |  |  |  |  |  |  |  |  |
| Farmer/worker |  |  |  |  | 1.00 (Reference) |  |  |  |  | 1.00 (Reference) |
| Government employee/technical professional | 0.28 | 0.06 | 22.47 | <.001 | 1.33 (1.18 ~ 1.49) | 0.13 | 0.07 | 3.20 | 0.074 | 1.14 (0.99 ~ 1.30) |
| Commercial/service worker | 0.17 | 0.08 | 5.34 | 0.021 | 1.19 (1.03 ~ 1.38) | 0.18 | 0.08 | 5.06 | 0.024 | 1.19 (1.02 ~ 1.39) |
| Retired | 0.18 | 0.08 | 5.57 | 0.018 | 1.19 (1.03 ~ 1.39) | 0.17 | 0.06 | 7.02 | 0.008 | 1.19 (1.05 ~ 1.35) |
| Other | 0.07 | 0.04 | 3.80 | 0.052 | 1.07 (1.00 ~ 1.15) | 0.22 | 0.05 | 17.89 | <.001 | 1.24 (1.12 ~ 1.38) |
| **Marital status** |  |  |  |  |  |  |  |  |  |  |
| Unmarried |  |  |  |  | 1.00 (Reference) |  |  |  |  | 1.00 (Reference) |
| Married | 0.02 | 0.06 | 0.11 | 0.739 | 1.02 (0.91 ~ 1.14) | -0.18 | 0.07 | 6.81 | 0.009 | 0.84 (0.73 ~ 0.96) |
| Divorced/widowed/other | -0.08 | 0.07 | 1.17 | 0.279 | 0.93 (0.81 ~ 1.06) | -0.36 | 0.09 | 15.05 | <.001 | 0.70 (0.58 ~ 0.84) |
| **Monthly personal income (CNY)** |  |  |  |  |  |  |  |  |  |  |
| 0–1499 |  |  |  |  | 1.00 (Reference) |  |  |  |  | 1.00 (Reference) |
| 1500–2999 | 0.12 | 0.03 | 13.62 | <.001 | 1.12 (1.06 ~ 1.19) | 0.03 | 0.06 | 0.31 | 0.575 | 1.03 (0.92 ~ 1.16) |
| 3000–4999 | 0.13 | 0.04 | 10.76 | 0.001 | 1.13 (1.05 ~ 1.22) | 0.16 | 0.06 | 8.07 | 0.004 | 1.17 (1.05 ~ 1.31) |
| 5000–7999 | 0.18 | 0.07 | 6.35 | 0.012 | 1.20 (1.04 ~ 1.38) | 0.16 | 0.07 | 4.88 | 0.027 | 1.17 (1.02 ~ 1.35) |
| ≥8000 | 0.01 | 0.14 | 0.00 | 0.944 | 1.01 (0.77 ~ 1.32) | -0.14 | 0.13 | 1.23 | 0.267 | 0.87 (0.67 ~ 1.12) |
| **Monthly household income (CNY)** |  |  |  |  |  |  |  |  |  |  |
| 0–2999 |  |  |  |  | 1.00 (Reference) |  |  |  |  | 1.00 (Reference) |
| 3000–5999 | 0.18 | 0.03 | 28.73 | <.001 | 1.19 (1.12 ~ 1.27) | 0.06 | 0.07 | 0.85 | 0.358 | 1.06 (0.93 ~ 1.21) |
| 6000–9999 | 0.28 | 0.04 | 51.84 | <.001 | 1.32 (1.22 ~ 1.42) | 0.16 | 0.07 | 5.81 | 0.016 | 1.17 (1.03 ~ 1.34) |
| 10000–19999 | 0.16 | 0.05 | 10.11 | 0.001 | 1.18 (1.06 ~ 1.30) | 0.27 | 0.07 | 14.59 | <.001 | 1.31 (1.14 ~ 1.51) |
| ≥20000 | 0.17 | 0.06 | 8.01 | 0.005 | 1.19 (1.05 ~ 1.33) | 0.28 | 0.08 | 10.63 | 0.001 | 1.32 (1.12 ~ 1.56) |
| **Alcohol use** |  |  |  |  |  |  |  |  |  |  |
| No |  |  |  |  | 1.00 (Reference) |  |  |  |  | 1.00 (Reference) |
| Yes | -0.14 | 0.03 | 22.28 | <.001 | 0.87 (0.82 ~ 0.92) | -0.12 | 0.05 | 5.66 | 0.017 | 0.88 (0.80 ~ 0.98) |
| **Smoking** |  |  |  |  |  |  |  |  |  |  |
| No |  |  |  |  | 1.00 (Reference) |  |  |  |  | 1.00 (Reference) |
| Yes | -0.06 | 0.03 | 3.96 | 0.047 | 0.94 (0.88 ~ 0.99) | -0.08 | 0.05 | 2.40 | 0.122 | 0.92 (0.83 ~ 1.02) |
| **SRH** |  |  |  |  |  |  |  |  |  |  |
| Good |  |  |  |  | 1.00 (Reference) |  |  |  |  | 1.00 (Reference) |
| Fair/poor | -0.39 | 0.03 | 148.84 | <.001 | 0.67 (0.63 ~ 0.72) | -0.62 | 0.06 | 123.65 | <.001 | 0.54 (0.48 ~ 0.60) |
| **Chronic disease** |  |  |  |  |  |  |  |  |  |  |
| No |  |  |  |  | 1.00 (Reference) |  |  |  |  | 1.00 (Reference) |
| Yes | -0.12 | 0.03 | 19.36 | <.001 | 0.88 (0.84 ~ 0.93) | -0.45 | 0.05 | 94.87 | <.001 | 0.64 (0.59 ~ 0.70) |

Notes: SRH,Self-rated health;MHL,Mental health literacy.

**Supplementary table 2.** Reference-coefficient sensitivity analysis of weighted Fairlie decomposition using urban sample coefficients

| **Terms of decomposition** | **Mental health literacy proficiency** | | | |
| --- | --- | --- | --- | --- |
| **Difference** | 0.0682249 | | | |
| **Explained (%)** | 0.0094396 (13.84%) | | | |
| **Non-explained (%)** | 0.0587852 (86.16%) | | | |
| **Explained** |  | | | |
| **Contribution to difference** | ***P*** | ***β*** | **Contribution (%)** | **[95%CI]** |
| Gender | <0.001 | 0.0005844 | 0.86 | (0.0005230, 0.0006290) |
| Region | <0.001 | -0.0276281 | -40.50 | (-0.0288710, -0.0259980) |
| Age group (years) | <0.001 | -0.0073085 | -10.71 | (-0.0080510, -0.0069070) |
| Education level | <0.001 | 0.0163360 | 23.94 | (0.0159090, 0.0169150) |
| Occupation | <0.001 | 0.0104929 | 15.38 | (0.0101130, 0.0108370) |
| Marital status | <0.001 | 0.0017872 | 2.62 | (0.0014650, 0.0022140) |
| Monthly personal income (CNY) | <0.001 | -0.0004083 | -0.60 | (-0.0006410, -0.0001890) |
| Monthly household income (CNY) | <0.001 | 0.0028943 | 4.24 | (0.0024430, 0.0031150) |
| Smoking | <0.001 | 0.0016410 | 2.41 | (0.0014590, 0.0017730) |
| Alcohol use | <0.001 | 0.0003205 | 0.47 | (0.0002550, 0.0003410) |
| SRH | <0.001 | 0.0055469 | 8.13 | (0.0046370, 0.0063160) |
| Chronic disease | <0.001 | 0.0051813 | 7.59 | (0.0044690, 0.0060650) |

Notes: SRH,Self-rated health;MHL,Mental health literacy.

**Supplementary table 3.** Reference-coefficient sensitivity analysis of weighted Fairlie decomposition using rural sample coefficients

| **Terms of decomposition** | **Mental health literacy proficiency** | | | |
| --- | --- | --- | --- | --- |
| **Difference** | 0.0682249 | | | |
| **Explained (%)** | -0.0212615 (-31.16%) | | | |
| **Non-explained (%)** | 0.0894864 (131.16%) | | | |
| **Explained** |  | | | |
| **Contribution to difference** | ***P*** | ***β*** | **Contribution (%)** | **[95%CI]** |
| Gender | <0.001 | -0.0006584 | -0.97 | (-0.0007220, -0.0006070) |
| Region | <0.001 | -0.0469760 | -68.85 | (-0.0480920, -0.0452720) |
| Age group (years) | <0.001 | -0.0024681 | -3.62 | (-0.0026700, -0.0019840) |
| Education level | <0.001 | 0.0092095 | 13.50 | (0.0088310, 0.0096190) |
| Occupation | <0.001 | 0.0109203 | 16.01 | (0.0104790, 0.0111320) |
| Marital status | <0.001 | 0.0006751 | 0.99 | (0.0005240, 0.0008720) |
| Monthly personal income (CNY) | <0.001 | -0.0027459 | -4.02 | (-0.0028040, -0.0025800) |
| Monthly household income (CNY) | <0.001 | 0.0052646 | 7.72 | (0.0048720, 0.0054580) |
| Smoking | <0.001 | 0.0025279 | 3.71 | (0.0021860, 0.0029130) |
| Alcohol use | <0.001 | 0.0000560 | 0.08 | (0.0000510, 0.0000690) |
| SRH | <0.001 | 0.0022692 | 3.33 | (0.0014630, 0.0026500) |
| Chronic disease | <0.001 | 0.0006643 | 0.97 | (0.0005610, 0.0007110) |

Notes: SRH,Self-rated health;MHL,Mental health literacy.
